# Changes, differences, and factors of parenthood in high-risk pregnant women and their partners in Japan

**DOI:** 10.1186/s12884-023-05519-3

**Published:** 2023-03-24

**Authors:** Eriko Kawamura, Midori Asano

**Affiliations:** Nagoya University Graduate School of Medicine, 1-1-20 Daiko Minami, Higashi-Ku, Nagoya, Aichi 461-8673 Japan

**Keywords:** High-risk pregnant women, Partners, Changes, Parenthood, Gender differences, Longitudinal study

## Abstract

**Background:**

Various stressors exists for pregnant women worldwide, especially negative social and environmental influences that can increase the number of high-risk pregnant women. These may cause a difficult transition to parenthood for women and their partners. However, limited studies have focused on and examined parenthood. Therefore, this study aimed to identify the changes in parenthood from pregnancy to post-discharge after childbirth among high-risk pregnant women and their partners, as well as the presence or absence of gender differences and the factors associated with parenthood.

**Methods:**

This longitudinal quantitative study used a self-administered anonymous questionnaire distributed among 127 pregnant women and their partners who visited a high-risk pregnant outpatient clinic. The Scale of Early Childrearing Parenthood (SECP; three subareas, 33 items) was administered thrice: during pregnancy (T1), after childbirth (T2), and after discharge (T3).

**Results:**

The analysis included 85 T1 (37 fathers and 48 mothers), 36 T2 (13 fathers and 23 mothers), and 31 T3 (11 fathers and 20 mothers) responses. There was a significant increase in the SECP scores for both parents from T1 to T3. Mothers had a greater increase in the SECP scores from T1 to T2 than fathers. In addition, fathers’ mean SECP scores at T1 and T2 were higher compared with those of the mothers. Mothers’ and fathers’ SECP scores at each time point showed no significant differences.

At all time points, the SECP scores were commonly and significantly associated with infertility treatment, physical and mental condition, postpartum depression at T2, and parenting stress at T3.

**Conclusions:**

Because parenthood in the infertility treatment group was significantly higher throughout the series, we need to support such couples so that childbirth does not become their main goal. We suggest interventions for factors that impede parenthood development, understand the various backgrounds of the parents, and support the couple individually while also considering them as a unit.

**Supplementary Information:**

The online version contains supplementary material available at 10.1186/s12884-023-05519-3.

## Background

For referencing purposes, the global total fertility rate (TFR) was forecasted to be 1.66 in 2100, and involved 23 countries, including Japan, Thailand, and Spain, forecasted to have a population decline of < 50% from 2017 to 2100, leading to severe population decline worldwide [1]. In Japan, the TFR is expected to be as low as 1.34 in 2020, and declining fertility is a major problem [2]. With the increase in the maternal age at delivery and the development of advanced reproductive medical technology, "high-risk pregnant women" with some risk of health problems, worsening complications, or death for both mother and child is increasing [3]. Specifically, the increasing trend toward late marriages has also increased reliance on reproductive healthcare, with 18.2% of couples currently undergoing testing and treatment for infertility [4]. Evidently, infertility is not a women-only problem, as approximately half of the causes of infertility rely on men [5]. In addition, the environment surrounding pregnant women today is likely to lead to increased isolation and the burden of child care [6, 7]. Particularly in high-risk pregnant women, such social, physical, and psychological factors as health concerns about themselves and their foetus may increase stress and difficulties in childcare. Since the coronavirus disease 2019 pandemic, fathers cannot be involved in childcare from birth until discharge due to limited access to medical facilities and experience increased stress in childcare [8–10]. Japan faces the pressing and important issues of declining fertility and childrearing difficulties. Among the various barriers, how the transition to parenthood relates to both men and women must be examined.

Thus, we focused on ‘parenthood’ among high-risk pregnant women and their partners to examine parental awareness and support in acquiring and developing their parenting competencies. Belsky [11] identified three parenting determinants: parental psychological resources, child characteristics, and contextual sources of support. Parenthood is a parenting role that is common to men and women and leads to improved parenting skills [12, 13]. It is characterized by love and respect for self as well as compassion and tenderness toward the child, which develops with the progression of life stages and is demonstrated in the protection and care of the child during pregnancy, delivery, and child rearing [13]. Pregnancy and the transition to parenthood are major adjustment periods for many adults, with important implications for new parents, couple relationships, and infant development [14]. Previous studies with high-risk pregnant women report postpartum depression (PPD) [15–17]; specifically, the association between depressed parents and parenting stress [18–20] and attachment and feelings toward the child [21, 22]. However, studies focusing on the parenthood of high-risk pregnant women are limited. Furthermore, studies focusing on fathers as partners are limited, and even fewer have identified differences in parenthood between mothers and fathers and the factors associated with parenthood.

Our objectives were as follows: (1) To examine how fathers’ and mothers’ parenthood changed from the gestational period to one month after the child’s discharge from the hospital and (2) to examine whether there were differences in parenthood between fathers and mothers during pregnancy, within seven days of delivery, and one-month post-discharge. Furthermore, we examined the factors associated with parenthood in each period. For mothers, parent–child attachment is formed during pregnancy [23], and while gaining a sense of togetherness through pregnancy, they can gradually develop affection for their children through the experience of delivery and breastfeeding [24]. Notably, paternal education does not include fathers, leading to a lack of preparation for parenthood for them [14]. Therefore, considering hypotheses for (1) and (2), we expect that changes in the development of parentage will be significantly greater for mothers. Furthermore, we expect that multiple factors are associated with the elements of parenthood [11–14], as indicated above since they are not composed solely of factors such as parenting ability and feelings toward the child.

These findings will aid nursing care that promotes the acquisition and development of parenthood from the early prenatal period. This may positively impact the child’s direct and indirect growth and development, as well as the parents’ growth and development. In addition, in clinical settings where there is a chronic lack of human resources and time limitations, validation of an effective parenthood development will contribute to the family support skills of healthcare workers and the well-being of children and their families. Furthermore, it is important to examine differences in the parenthood of mothers and fathers, as studying gender differences can lead to gender innovation [25].

## Methods

### Design

This longitudinal research study used a self-administered anonymous questionnaire survey.

### Participants and procedures

Participants were recruited from two high-risk pregnant women’s hospitals in Japan between April and December 2021. Pregnant women and their partners (Japanese) who visited the hospital were considered eligible to be candidates for research collaboration by the outpatient physician, and those who agreed to cooperate were recruited as participants. We excluded pregnant women’s partners who were not related to the foetus.

We requested the target facilities’ co-operation for this study and explained the study in writing and orally. After consent was obtained from the facilities, approval was obtained from the ethical review committee of each facility. Subsequently, we asked the attending physicians in the outpatient clinics to list the potential participants, which are all couples who fulfilled the above criterion.

Finally, the researcher explained the study in writing and orally to the patients who agreed to participate, obtained their consent, and gave them a questionnaire to complete anonymously.

Participants completed a questionnaire at three-time points: during pregnancy (Time point 1 [T1]), within seven days of delivery (Time point 2 [T2]), and within one month after the child’s discharge (Time point 3 [T3]), and mailed it each time.

### Questionnaires

#### Participant background

We surveyed 24 items divided into three categories of participant backgrounds. The items were as follows: (1) Parents’ Characteristics: gender, age, job status, education level, family patterns, years of marriage, infertility treatment, number of children, pregnancy weeks at the time of the survey, mental and physical conditions, and fostered environment; (2) Child characteristics: birth weeks, birth weight, sex, multiple births, NICU hospitalisation, and length of hospitalisation; (3) Childcare and housework: child handling experience, anxiety for childcare, anxiety except for childcare, husbands work- wives do housework, male participation in childcare, childcare leave, and participation in childcare during hospitalisation.

#### Measurement Scales

The roots used in this study are shown in Table 1. The Scale of Early Childrearing Parenthood (SECP) [26, 27] was administered at T1, T2, and T3. The scale was developed by Ohashi and Asano [26] based on the maternal role attainment theory of Mercer [28], and its reliability and validity have been confirmed. The SECP has been increasingly used in Japan in recent years. The SECP assessed parental qualities and divided them into awareness of the child and self-awareness. Awareness of oneself comprised the state of the parental and non-parental roles, combined with an awareness of the child, to form the scale’s three subdomains. This self-administered questionnaire consisted of 33 items: 13, 9, and 11 items on the state of the parental role, non-parental role, and awareness of the child, respectively. A high total score indicated high awareness of oneself and the child, and there was no cut-off value.

**Table 1:** Tools in use

Tool Name	Number of Items	Subdomains（Number of Items）	Answer Format	Cronbach’s α
SECP	33	The state of the parental role(13)	5-point Likert scale	0.82 -0.94
		The state of the non-parental role(9)		
		Awareness of the child(11)		
EPDS	10	–	4-point scale	0.82
PSI-SF	19	Parent(10)	5-point Likert scale	0.66 - 0.88
		Child(9)		

The state of the parental role comprised parental satisfaction, concern for the child, and the relationship with the child. The state of the non-parental role comprised feelings of satisfaction with self-independence as a parent, self-acceptance, and relationship with society. Awareness of the child covered affection towards the child, understanding of the child and their developmental growth, and child-rearing ability. The items were rated on a five-point Likert scale with the following response options: strongly agree, agree, neither, disagree, and strongly disagree. The total scores were calculated by adding each subscale’s scores, ranging from 33 to 165. Cronbach’s alpha for the SECP in the present study ranged from 0.82 to 0.94.

The Edinburgh Postnatal Depression Scale (EPDS) [29] was administered at T2. The Japanese version of the EPDS [30], originally developed by Cox, Holden, and Sagovsky [28], assessed PPD. This self-administered questionnaire consisted of ten items, each scored on a four-point scale (0–3). The scale’s reliability and validity have been well established [30]. If the total score was ≥ 9 points, or if the question item ‘I have had thoughts of hurting myself’ was ≥ 1 point, it was screened as possibly depressed. Cronbach’s alpha for the EPDS in this study was 0.82.

The Parenting Stress Index-Short Form Scale (PSI-SF) [31] was administered at T3. The Japanese version of the PSI-SF [31], originally developed by Abidin [32] and based on the full-length PSI [33], was used to assess parenting stress. This scale has been widely used, both nationally and internationally. This self-administered questionnaire consisted of 19 items and two subdomains: ten and nine items on the parent and child, respectively. The items were rated on a five-point Likert scale with the following response options: strongly agree, agree, neither, disagree, and strongly disagree. Higher scores indicated greater parenting stress. The total scores were calculated by adding each subscale’s scores, ranging from 19 to 95. The scale’s reliability and validity have been well established [29]. Cronbach’s alpha for the PSI-SF in the present study ranged from 0.66 to 0.88.

### Statistical analysis

Descriptive statistics were calculated to provide an overview of the participants. Descriptive data were expressed as means (range) for continuous variables and the number of persons (%) for nominal variables.

First, a paired t-test was performed to determine the change in the fathers’ and mothers’ SECP total and three subscale scores during T1, T2, and T3. Next, a t-test was conducted to determine the differences between the fathers’ and mothers’ SECP total and the three subscale scores at T1, T2, and T3. Finally, to identify the factors associated with the SECP at T1, T2, and T3, t-tests were conducted to compare two categories (e.g., father vs. mother), Pearson’s product-moment correlation analysis for continuous variables (e.g., age, EPDS, and PSI-SF), while one-way analysis of variance was used with more than two categories (e.g., job status). Assumptions of normality were evaluated. For items that showed significant differences from the SECP, Cohen’s d was calculated as an effect size to clarify the size of the difference in substantive means [34]. Effect sizes of 0.20, 0.50, and 0.80 were considered small, moderate, and large, respectively [34]. The criterion for statistical significance was set at a two-sided significance probability of less than 5%. All statistical analyses were performed using SPSS (IBM SPSS Statistics 28 for Windows, Tokyo, Japan).

This study was approved by the Bioethics Review Committee of the Nagoya University Graduate School of Medicine (2020–0322) and Ogaki municipal hospital Institutional Review Board (20,200,528–1). All methods were carried out in accordance with relevant guidelines and regulations (such as the Declaration of Helsinki).

## Results

**Fig. 1:**
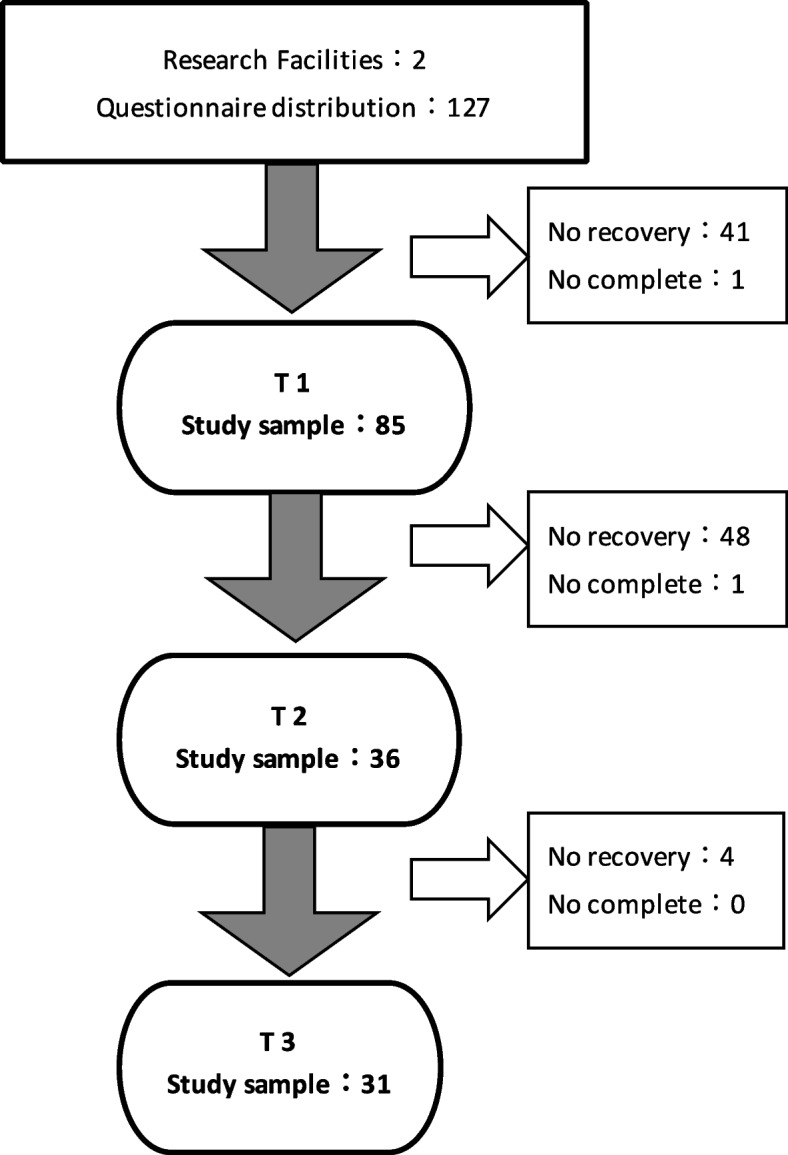
Flow chart of participants. High-risk pregnant women who visit the research facilities are defined as those who may be at some risk of health problems, worsening complications, or death for both mother and child during pregnancy, childbirth, or after delivery

The recruitment process is shown in Fig. 1. In total, 127 questionnaires were distributed. Of these, 86 (67.7%) responses were collected, 85 (98.8%) were valid at T1, 37 (29.1%) were collected, 36 (28.3%) were valid at T2, and 31 (24.4%) valid responses were collected at T3.

### Participants’ background

Table 2 shows the background information of each participant at T1, T2, and T3. In T1, more than half (48/85, 56.5%) in T1, more than 60% (23/36, 63.9%) in T2, and more than 60% (20/31, 64.5%) in T3 were mothers. Approximately half (44/85, 51.8%) were the first child in T1, less than half (17/36, 47.2%) in T2, and approximately half (16/31, 47.2%) in T3 were the first child. One quarter (9/36, 25.0%) were screened for risk of PPD (hereafter referred to as EPDS positive) and admitted to the NICU in T1 and T2, respectively, and less than 20% (5/31, 25.0%) were admitted to the NICU in T3. The mean PSI-SF scores were 37.77 (22–55) total points.

**Table 2:** Participants’ Background

	T1^a^ (*n*=85)		T2^a^ (*n*=36)		T3^a^ (*n*=31)	
	n(%)	M(range)	n(%)	M(range)	n(%)	M(range)
Parents'Characteristics						
Gender						
Father	37(43.5)		13(36.1)		11(35.5)	
Mother	48(56.5)		23(63.9)		20(64.5)	
Age（years old)		32.61(21-50)		33.94(22-50)		33.84(22-50)
Job Status ^b^						
Regular	54(63.5)		22(61.1)		20(64.5)	
Non-regular	9(10.6)		5(13.9)		3( 9.7)	
Self-employment	4( 4.7)		2( 5.6)		2( 6.4)	
Unemployed	18(21.2)		7(19.4)		6(19.4)	
Education Level						
Less than high School diploma	22(25.9)		9(25.0)		7(22.6)	
College/University or higher	63(74.1)		27(75.0)		24(77.4)	
Family Patterns						
Nuclear	75(88.2)		32(88.9)		29(93.5)	
Extend	10(11.8)		4(11.1)		2( 6.5)	
Years of Marriage						
Less than 3 years	35(41.2)		12(33.3)		11(35.5)	
More than 3 years	50(58.8)		24(66.7)		20(64.5)	
Infertility Treatment ^c^						
Yes	29(34.1)		11(30.6)		9(29.0)	
Non-regular	56(65.9)		25(69.4)		22(71.0)	
Number of Children						
First child	44(51.8)		17(47.2)		16(51.6)	
Second child or more	41(48.2)		19(52.8)		15(48.4)	
Pregnancy Weeks at Time of Survey						
Less than 28 weeks	31(36.5)		−		−	
More than 28 weeks	54(63.5)		−		−	
Mental and Physical Condition						
Both in good condition	65(76.5)		26(72.2)		21(67.7)	
Either or both in poor condition	20(23.5)		10(27.8)		10(32.3)	
Fostered Environment						
Attachment	82(96.5)		34(94.4)		30(96.8)	
No attachment	3( 3.5)		2(5.6)		1( 3.2)	
Child Characteristics						
Birth Weeks (weeks)				37.78(26-41)		37.87(26-41)
Birth Weight						
Less than 2500g	−		9(25.0)		6(19.4)	
More than 2500g	−		27(75.0)		25(80.6)	
Sex						
Boy	−		21(58.3)		19(61.3)	
Girl	−		15(41.7)		12(38.7)	
Multiple Births						
Yes	−		3( 8.3)		0( 0.0)	
No	−		33(91.7)		31(100.0)	
NICU Hospitalization						
Yes	−		9(25.0)		5(16.1)	
No	−		27(75.0)		26(83.9)	
Length of Hospitalization						
Less than 1 week	−		−		23(74.2)	
From 1 week to 1 month	−		−		5(16.1)	
More than 1 month	−		−		3( 9.7)	
Childcare and Housework						
Child Handling Experience						
Yes	63(74.1)		30(83.3)		25(80.6)	
No	22(25.9)		6(16.7)		6(19.4)	
Anxiety for Childcare						
Yes	40(47.1)		13(36.1)		13(41.9)	
No	45(52.9)		23(63.9)		18(58.1)	
Anxiety Except for Childcare						
Yes	21(24.7)		6(16.7)		6(19.4)	
No	64(78.3)		30(83.3)		25(80.6)	
Husbands Work, Wives Do Housework						
Agree	14(16.5)		8(22.2)		4(12.9)	
Against	71(83.5)		28(77.8)		27(87.1)	
Male Participation in Childcare						
Very much agree	66(77.6)		28(77.8)		24(77.4)	
More or less agree	19(22.4)		8(22.2)		7(22.6)	
Childcare Leave						
Yes	−		19(52.8)		18(58.1)	
No	−		17(47.2)		13(41.9)	
Participation in Childcare during Hospitalization						
Yes	−		−		28(90.3)	
No	−		−		3( 9.7)	
EPDS						
Score		−		4.75(0-14)		−
Negative	−		27(75.0)		−	
Positive	−		9(25.0)		−	
PSI-SF						
Overall Score		−		−		37.77(22-55)
Child Aspects		−		−		18.84( 9-27)
Parent Aspects		−		−		18.94(10-30)

^a^"T" is the assessment time point, where T1 is during pregnancy, T2 is seven days after delivery, and T3 is within one month after the child’s discharge

^b^Regular employment includes company employees and civil servants. Unemployed include housewives and househusbands

^c^Fertility treatment includes various methods such as hormonal therapy, timing methods, AIH (Artificial insemination), IVF (In vitro fertilisation), and ICSI (intra-cytoplasmic sperm injection)

The data analysis addressed the three major aims. First, we addressed how father-mother parenthood changed from pregnancy to post-discharge of the infants. We also examined whether fathers and mothers had differences in parenthood during the three-time points of pregnancy, postpartum, and post-discharge. The factors associated with parenthood at each time point were also examined.

### Changes in parenthood for fathers and mothers

The 31 participants who participated in all three periods (fathers, 11; mothers, 20) were included in the analysis. Changes in the SECP scores for fathers and mothers from T1 to T3 are shown in Table 3 (Supplemental Fig. S1- a, b, c, and d).

**Table 3:** Comparison of SECP scores across assessment times for father and mother

	Overall Scores					State of the Parental Role				State of the Non-Parental Role				Awareness of the Child				
	M (SD)	T1		T2		M (SD)	T1		T2	M (SD)	T1		T2	M (SD)	T1		T2	
		p	d	p	d		p	d	p		p	d	p		p	d	p	d
All（*n*=31）																		
T1	112.74(13.12)	−	−	−	−	48.65(6.04)	−	−	−	32.06(5.90)	−	−	−	32.03(6.66)	−	−	−	−
T2	125.48(13.52)	＜0.001	1.02	−	−	54.55(6.22)	＜0.001	1.02	−	34.13(5.13)	0.022	0.44	−	37.13(7.44)	＜0.001	0.90	−	−
T3	129.48(15.46)	＜0.001	1.08	0.037	0.390	55.74(6.39)	＜0.001	1.05	0.210	34.58(5.88)	0.003	0.58	0.379	39.32(6.98)	＜0.001	0.93	0.030	0.41
Father（*n*=11）																		
T1	117.18(11.32)	−	−	−	−	49.55(6.44)	−	−	−	33.36(4.70)	−	−	−	34.27(6.48)	−	−	−	−
T2	125.82(11.55)	0.029	0.77	−	−	52.27(6.62)	0.052	−	−	34.18(5.40)	0.624	−	−	39.36(6.27)	0.017	0.87	−	−
T3	129.00(11.03)	0.025	0.79	0.282	−	54.55(6.50)	0.023	0.79	0.131	35.09(5.59)	0.271	−	0.356	39.09(5.75)	0.044	0.69	0.841	−
Mother（*n*=20）																		
T1	110.30(13.66)	−	−	−	−	48.15(5.91)	−	−	−	31.35(6.47)	−	−	−	30.80(6.58)	−	−	−	−
T2	125.30(14.45)	＜0.001	1.17	−		55.80(5.78)	＜0.001	1.30	−	34.10(5.12)	0.011	0.63		35.90(7.89)	＜0.001	0.89	−	−
T3	130.00(18.17)	＜0.001	1.21	0.068	−	56.40(6.39)	＜0.001	1.19	0.632	34.30(6.15)	0.050	0.72	0.743	39.45(7.71)	＜0.001	1.07	0.01	0.66

The statistical method is a paired T-test

From T1 to T2, the ‘overall score’ and ‘awareness of the child’ increased significantly for both fathers and mothers by more than moderate margins. In contrast, the ‘state of the parental role’ and the ‘state of the non-parental role’ increased significantly by more than moderate margins only for mothers.

From T2 to T3, ‘overall score,’ ‘state of the parental role,’ and ‘state of the non-parental role’ did not increase significantly for either parent. However, ‘awareness of the child’ increased significantly by a moderate margin only for mothers.

From T1 to T3, the ‘overall score,’ ‘state of the parental role,’ and ‘awareness of the child’ increased significantly for both parents, with more than moderate differences.

In contrast, the ‘state of the non-parental role’ increased only for mothers by moderate margins, while fathers did not change.

### Differences in parenthood between fathers and mothers

The details are presented in Table 4. The mean SECP scores at T1, T2, and T3 were not significantly different between parents for ‘overall score’ and all three subscale scores.

**Table 4:** Differences in SECP scores between father and mother at each assessment time-point

	Overall Scores		State of the Parental Role		State of the Non-Parental Role		Awareness of the Children	
	M (SD)	p	M (SD)	p	M (SD)	p	M (SD)	p
T1(*n*=85)								
Father	120.68(15.83)	0.486	50.27(6.82)	0.462	34.03(5.57)	0.167	36.38(7.97)	0.311
Mother	118.15(17.04)		51.35(6.61)		32.17(6.48)		34.63(7.70)	
T2(*n*=36)								
Father	125.62(10.60)	0.976	52.31(6.07)	0.084	34.08(5.14)	0.850	39.23(5.82)	0.239
Mother	125.48(13.86)		55.83(5.49)		33.74(5.08)		36.35(7.64)	
T3(*n*=31)								
Father	129.00(11.03)	0.900	54.55(6.50)	0.449	35.09(5.59)	0.726	39.09(5.75)	0.897
Mother	130.00(18.17)		56.40(6.39)		34.30(6.15)		39.45(7.71)	

The statistical method is a T-test; *SD* Standard deviation, *M* Mean

### Factors related to parenthood

Table 5 shows the overall SECP scores and associated factors at each assessment. The following two items significantly differed from the total SECP scores for T1, T2, and T3: infertility treatment (higher score for ‘yes’) and mental and physical condition (higher score for ‘both in good condition’).

**Table 5:** Factors associated with SECP (overall score) at each assessment time point

All	T1			T2			T3		
	M (SD)	p	d	M (SD)	p	d	M (SD)	p	d
	r			r			r		
	119.25(16.48)			125.53(12.62)			129.48(15.46)		
Infertility Treatment^a^									
Yes	126.69(15.45)	0.002	0.72	132.82(14.37)	0.019	0.89	140.78(14.21)	0.007	1.15
Non-regular	115.39(15.77)			122.32(10.54)			124.86(13.69)		
Pregnancy Weeks at Time of Surve^a^									
Less than 28 weeks	125.32(15.14)	0.005	0.61	−			−		
More than 28 weeks	115.76(16.32)			−			−		
Mental and Physical Condition^a^									
Both in good condition	121.97(15.98)	0.005	0.73	128.42(12.69)	0.024	0.88	133.95(14.03)	0.017	0.97
Either or both in poor condition	110.40(15.25)			118.00( 9.12)			120.10(14.63)		
Anxiety for Childcare^a^									
Yes	113.88(17.36)	0.004	0.66	120.08(10.10)	0.050	−	123.78(14.25)	0.191	−
No	124.02(14.20)			128.61(13.05)			132.05(16.09)		
Anxiety Except for Childcare^a^									
Yes	110.81(16.34)	0.006	0.71	121.17( 9.50)	0.361	−	120.33(20.75)	0.107	−
No	122.02(16.67)			126.40(13.11)			131.68(13.52)		
EPDS^b^									
Score	−			-0.43	0.009		−		
PSI-SF^b^									
Overall Score	−			−			-0.65	＜0.001	
Child Aspects	−			−			-0.57	＜0.001	
Parent Aspects	−			−			-0.59	＜0.001	

^a^T-test; ^b^Pearson product-moment correlation analysis; *SD* Standard deviation, *M* Mean, *EPDS* The Edinburgh Postnatal Depression Scale, *PSI-SF* The Parenting Stress Index-Short Form Scale

For T1, three additional items were significantly different from the SECP: Pregnancy weeks at the time of the survey (higher score for ‘less than 28 weeks’), anxiety regarding childcare (higher score for ‘no’), and anxiety, except for childcare (higher score for ‘no’).

For T2, the EPDS (negative correlation) and for T3, the PSI-SF (negative correlation for both the overall score and scores on the children’s and parents’ aspects) were significantly different from the SECP.

## Discussion

This is the first study to measure parenthood from pregnancy for high-risk pregnant women and their partners, which discussed the changes in parenthood and the associated factors for fathers and mothers.

In this study, both fathers’ and mothers’ SECP scores increased significantly from pregnancy till the child’s discharge. During pregnancy, the SECP scores for mothers were lower than for fathers. However, the SECP scores for mothers increased significantly after childbirth, especially in the ‘state of the parental role,’ which was considerably higher than that of fathers. This may be because of the mothers’ experiences of childbirth. The results of the present study were consistent with the fact that the development of parenthood towards infants was more pronounced in mothers than in fathers and that mothers who were more involved in child-rearing underwent greater changes as a result of becoming parents [35]. Contrary to the hypothesis, an interesting finding of this study was that the mean SECP scores (which excluded ‘state of the parental role’) during pregnancy and after birth were higher for fathers than for mothers. Although it was generally believed that mothers were more parental than fathers [36], the opposite was true in this study. This may be characteristic of high-risk pregnant couples. The promotion of communication between the couple during the pregnancy [37, 38] and the enhancement of support for the marital relationship [39] have contributed to the men’s adjustment to fatherhood. In contrast, wives perceived parenthood as more constraining and burdensome than their husbands, which may be because wives experienced pregnancy and childbirth, undertook physiological changes, and were subject to actual constraints [40]. In particular, it has been reported that the mother’s illness and severe fatigue in older primiparas were related to higher parental stress two months after childbirth [41]. In this study, the fathers’ parenthood was higher than that of the mothers, possibly because the couples had communicated and solved various problems before the child’s birth. Furthermore, high-risk pregnant women were especially subject to greater physical and emotional strain than the general pregnant population.

There was no significant difference in the SECP scores between high-risk pregnant women and their partners during pregnancy, after childbirth, and after discharge, which indicated no gender difference in parenthood before and after childbirth. This result was consistent with previous studies that showed no gender differences in SECP scores from immediately after childbirth to the early parenting period [42, 43]. Thus, both fathers and mothers followed similar parental transition processes. However, these results were obtained only after childbirth. This result indicated no gender differences in parenthood during pregnancy or even before birth, which was a novel finding.

Finally, we found that fertility treatment and mental and physical conditions were commonly associated with the parenthood of high-risk pregnant women and their partners throughout the continuum from pregnancy to their child’s discharge. In addition, their parenthood was also related to the EPDS score after childbirth and the PSI-SF score after the child’s discharge.

The infertility treatment rate for our participants in this study was > 30%, which was higher than that of the general group. Women who conceived and gave birth after infertility treatment viewed their infertility experiences as meaningful [44]. Furthermore, for patients who underwent infertility treatment, ‘having a child’ was sometimes described as a goal to be fulfilled [45]. The increased self-esteem gained from fulfilling goals may have influenced the parenthood of infertility-treated couples. In addition, the shared stress of infertility may even stabilize marital relationships [46–48], resulting in increased parenthood. This study showed significantly higher SECP scores in the infertility treatment group at all time points from conception to post-discharge. However, it cannot be denied that childbirth tended to be their goal. Most studies indicated no difference in anxiety regarding pregnancy, foetal development, and delivery between those who underwent fertility treatment and those who conceived naturally. However, when the items were examined, there were some differences, and the results were inconsistent [49]. In addition, in the infertility experience, overall, people were more likely to experience poorer well-being (e.g., higher depression and negative affect) when they faced a blocked parenthood goal [50]. An examination of the congruence between partners’ perceived infertility-related stress and its relationship to marital adjustment and depression in infertile couples showed that couple incongruence was unrelated to depression in males and incongruence over relationship concerns. Furthermore, the need for parenthood was related to female depression [46]. Additionally, studies indicated that partners who underwent in vitro fertilization (IVF) might not have enough support from their closest social environments [51, 52]. Previous research has shown that infertility is stressful. Thus, while infertility experience may strengthen the couple’s bonds, it increases the stress caused by the vulnerability of support. Hence, whether infertility experience directly affects the development of parenthood and how parenthood might change should be examined.

The results of mental and physical conditions were similar to those of previous studies [53]. The support for mental health is important, especially since the EPDS and parenting stress were relevant for postpartum parenthood. Recently, PPD in fathers and mothers has been increasing, and its prevalence was not significantly different between men and women [54]. Paternal PPD is associated with relationships and physical health and has negative effects on children [55, 56]. Furthermore, it is also associated with PPD in mothers, and there was concern that the child-rearing environment may deteriorate if the couple suffered from mental illness simultaneously [55]. In addition, parenting stress more significantly affects anxiety than anger [57]. Therefore, physical and mental conditions and depressive symptoms, including parenting stress, should be considered together.

In the present study, only a few children were admitted to the NICU, who were low birth-weight babies. Japan has drastically reduced rates of maternal, perinatal, and neonatal or infant deaths, making it the safest country to give birth and raise a child [3]. Thus, the results indicate that the high-risk pregnant outpatient clinic functioned effectively and avoided risk. Moreover, there were no significant differences between parenthood and child factors. However, the child’s health was a major concern for high-risk pregnant women and their partners. According to previous studies, the risk of parental PPD was four to eighteen and three to nine times higher for mothers and fathers of VLBW infants, respectively, compared with mothers and fathers of term infants [16]. Therefore, it is necessary to emphasize the importance of assessing the parents’ mental state and the child’s health problems and providing nursing interventions.

### Practical implications

We suggest that couples treated for infertility in high-risk pregnant outpatient clinics require support so that childbirth does not become the goal and that parenthood, which increases through childbirth, does not decrease. For mothers, there is a need to intervene with an awareness of the importance of birth review as the delivery experience and immediate contact with the child may affect subsequent parenthood. Prior research showed that fathers perceived that perinatal health professionals viewed ‘mothers as a priority’ [58]. Thus, we suggest interventions that stimulate fathers with experiences that increase their awareness of parenthood, especially during the transition to parenthood before and after birth, since, unlike mothers, they undergo fewer physical changes. Furthermore, hospitalized high-risk obstetrical patients may commonly experience depression or anxiety symptoms and not receive treatment [59] and, therefore, may not be intervening despite the predicted high-risk factors.

As described above, parenthood may be impeded without third-party intervention for high-risk pregnant women and their partners. Therefore, we believe that healthcare providers must be aware of these issues and recognize the requirement for long-term involvement in individual problems, considering the various backgrounds of pregnant women and their partners. In particular, mental health assessments during the postpartum period should be promoted for both mothers and fathers to prevent parental psychological distress [60]. We suggest viewing parents as one unit rather than separate, as couples interacting with each other regarding mental health issues.

Finally, a significant solution to the global population decline, including Japan, is sustaining and enhancing female reproductive health [1]. The main target groups of reproductive health care are women, mothers, foetuses, and children, but it also includes men as reproductive and child-rearing partners [61]. Therefore, ongoing support for women and their partners, starting at the stage of fertility treatment prior to pregnancy, will contribute to the decline in fertility.

### Strengths and limitations

The strength of this study was that although support for expectant mothers was strengthened and focused on ‘seamless support’ from pregnancy, data on fathers were valuable in emphasizing the need for support. Including both fathers and mothers provided a more dynamic view of the family system. In addition, including data from the pregnancy period was novel as data using the SECP only covered the period from birth to the first year of life. Furthermore, this study added to the knowledge of parents with a history of infertility treatment. In Japan, the government started a system to provide universal health insurance coverage for infertility treatment in 2022 [62]. Since this demand is expected to increase in modern society, this study will support couples who overcome difficulties in becoming new parents.

This study has some limitations. First, the sample size was inadequate due to the rarity of the participants. During the survey period, Japan fell into the seventh wave of the coronavirus disease 2019 (COVID-19) pandemic, and direct recruitment has been impossible since because of the ban on researchers entering and leaving the facilities. Moreover, for the reasons stated above, the inability to directly remind participants at the time of delivery may have been the reason for the significant decrease in frequency rates during the T2 phase. Hence, the possibility that some items failed to show significant differences due to the insufficient sample size cannot be ruled out. In particular, the small number of treatment groups in the NICU was unexpected, and this requires further examination.

Second, in Japan, there is still an underlying cultural value of "men work, women raise children," a gender role division of labour. Therefore, these values may affect parenthood and may not be transferable to other cultures in other countries worldwide. This study focused on Japanese nationals; of all new-borns in Japan in 2017, less than 3% had a foreign mother, a 26% increase from the previous two decades [63]. Furthermore, this number is expected to increase. Therefore, to realize seamless support for all pregnant women giving birth in Japan, data from foreign-born participants are required, and must be considered by future studies.

## Conclusion

In this study, there was no difference in the parenthood of high-risk pregnant women and their partners, but the father's parenthood was higher than the mother's during pregnancy and after childbirth. Throughout the series, from pregnancy to discharge, parenthood was commonly associated with infertility treatment and physical and mental conditions.

The parenthood of the fertility treatment group was significantly higher during pregnancy, after delivery, and post-discharge. Hence, for couples who received infertility treatment at high-risk pregnancy outpatient clinics, we suggest interventions for factors that impede parenthood development, understand the various backgrounds of the participants, and provide individualised long-term support so that childbirth does not become the goal of the couple’s life. In particular, this study suggests the need to support postpartum mental health by considering couples as a unit.

## Supplementary Information

**Additional file 1: Figure S1.** Changes in the SECP scores for fathers and mothers. Blue lines and letters represent fathers; red lines and letters represent mothers. Each represents ***p<0.001 **p<0.01 *p<0.05. The ‘d’ in the figure indicates the effect size.

## Data Availability

A de-identified dataset of the quantitative survey responses analysed during the current study is available from the corresponding author upon reasonable request.

## Abbreviations

TFR: Total fertility rate
NICU: Neonatal intensive care unit
PPD: Postpartum depression
VLBW: Very low birth weight
Time point 1: T1
Time point 2: T2
Time point 3: T3
SECP: Scale of Early Childrearing Parenthood
EPDS: Edinburgh Postnatal Depression Scale
PSI-SF: Parenting Stress Index–Short Form scale
IVF: In vitro fertilisation
COVID-19: Coronavirus disease 2019
