# Supplementary material for: Changes, differences, and factors of parenthood in high-risk pregnant women and their partners in Japan
Source: BMC Pregnancy Childbirth. 2023 Mar 24;23:205. doi: 10.1186/s12884-023-05519-3 (PMC10037369; doi:10.1186/s12884-023-05519-3)
Supplement: Supplementary file 1 — Additional file 1: Figure S1. Changes in the SECP scores for fathers and mothers. Blue lines and letters represent fathers; red lines and letters represent mothers. Each represents ***p<0.001 **p<0.01 *p<0.05. The ‘d’ in the figure indicates the effect size. [file 12884_2023_5519_MOESM1_ESM.docx]

a )

b )

c )

d )

Figure S1 Changes in the SECP scores for fathers and mothers

Blue lines and letters represent fathers; red lines and letters represent mothers. Each represents ***p<0.001 **p<0.01 *p<0.05. The 'd' in the figure indicates the effect size.
